# Supplementary material for: Muscle5: High-accuracy alignment ensembles enable unbiased assessments of sequence homology and phylogeny
Source: Nat Commun. 2022 Nov 15;13:6968. doi: 10.1038/s41467-022-34630-w (PMC9664440; doi:10.1038/s41467-022-34630-w)
Supplement: Supplementary file 1 — Supplementary Information [file 41467_2022_34630_MOESM1_ESM.pdf]

Muscle5: High-accuracy alignment ensembles enable unbiased assessments of sequence homology and phylogeny

Robert C. Edgar

Supplementary Methods

Muscle5 algorithm

Here I provide further details of the algorithms with motivation and comparison with prior work. The primary algorithm component of `Muscle5` is `PPP`, a re-implementation of `ProbCons` with improvements including parallelisation, parameter perturbations and support for both amino acid and nucleotide sequences. Datasets of up to a few hundred sequences are aligned by `PPP`, larger datasets are aligned by `Super5` (see below) which applies a divide-and-conquer strategy to `PPP`.

Parallel Perturbed ProbCons (PPP)

`ProbCons` generalises the pair-wise posterior decoding alignment algorithm [1] to protein multiple alignment, applying a consistency transformation to the posterior probability matrices. Posteriors are calculated using the hidden Markov model (HMM) shown in Fig. S1. Since publication, `ProbCons` has remained among the top-scoring methods on protein alignment benchmarks with small numbers of sequences, but is computationally expensive, scaling to at most a few tens of sequences on a current commodity computer, and does not support nucleotide sequences. Here, a commodity computer is operationally defined as a `c5a.4xlarge` instance on Amazon Web Services (`https://aws.amazon.com/`), which has 16 vCPU cores and 32 Gb of RAM. In `PPP`, calculation of the pair-wise posterior probability matrices and the consistency transformation are parallelised, enabling alignment of hundreds of sequences in times ranging from minutes up to a few hours on a `c5a.4xlarge`.

PPP profile alignment (PPP-pa)

The final stage of `PPP` performs progressive alignment where a pair X, Y of MSAs with associated posterior probabilities (profiles) is aligned by maximising the total posterior probability under the constraint that columns in each MSA are held fixed. This is achieved by calculating a matrix M as follows [2],

$$M_{ij} = \sum_{x \in X} \sum_{y \in Y} P(x_i \leftrightarrow y_j), \tag{1}$$

where x is a sequence in MSA X, y is a sequence in MSA Y, and $P(x_i \leftrightarrow y_j)$ is the posterior probability that the letter of x in column i of X aligns to the letter of y in column j of Y. In

PPP, this calculation is parallelized by observing that the contributions to M_{ij} from different pairs of sequences in Eq. 1 are independent, and can therefore be calculated on separate threads. The profile alignment is determined by using dynamic programming to maximise the sum of M_{ij} over alignment columns. I call this method for aligning a pair of profiles PPP-pa.

PPP profile alignment with subsampling (PPP-ps)

PPP-pa has complexity $O(N_X N_Y)$ in the number of sequences N_X and N_Y in X and Y respectively. For large N, this cost can be prohibitive. Super5 implements a faster approximation to PPP-pa by selecting random subsets X^* and Y^* of the sequences in X and Y respectively. The subsampled matrix M^* is then computed as follows,

$$M^*_{ij} = \sum_{x \in X^*} \sum_{y \in Y^*} P(x_i \leftrightarrow y_j). \tag{2}$$

The maximum posterior alignment is then calculated using M^* rather than M. I call this method PPP-ps. Super5 aligns profiles using PPP-pa if $N_X N_Y \leq n_{max}$ (default $n_{max} = 2,000$), otherwise a random subset of sequence pairs is selected and PPP-ps is used.

Expected-error distance

The expected error rate (fraction of incorrect columns) in the posterior decoding alignment A of sequences x and y is calculated as follows [1],

$$EE = 1 - \frac{1}{|A|} \sum_{i,j \in A} P(x_i \leftrightarrow y_j). \tag{3}$$

Sequences that can be more accurately aligned (according to the HMM) have smaller EE, which can be considered as an approximate distance measure defined on pairs of sequences (it is not strictly a distance because the triangle inequality is not necessarily satisfied). Assuming that EE correctly predicts accuracy, the total number of errors can be greedily minimised by aligning the closest pair at each iteration, i.e. by progressive alignment using a UPGMA guide tree. Both PPP and Super5 construct UPGMA guide trees using EE distances. Super5 uses EE-based

clustering to reduce redundancy and divide sequences into smaller sets that are tractable for `PPP` (described in more detail below).

Guide tree variants

Both `PPP` and `Super5` use progressive alignment. Variant guide trees are constructed as follows. The goal is to identify three large subtrees a, b, c which are joined in all three possible orders $((a, b), c)$, $((a, c), b)$ and $((b, c), a)$. This is achieved by considering all possible bifurcations of the original tree (which is temporarily considered to be unrooted), and identifying the edge which most closely approximates dividing the tree into subtrees with one third and two thirds of the sequences, respectively. The smaller subtree is a. The larger subtree bc is divided into two equal-sized (or approximately equal-sized) subtrees b and c by a similar search for the best edge, as shown in Fig. S2. Including the original guide tree, this gives a total of four variant guide trees for generating ensembles.

This design accomplishes a number of objectives. Close to the root, there should be substantial variations in the joining order of large groups to induce substantive variations into any bias caused by progressive alignment. Close to the leaves, the joining order should be preserved as far as possible because alignment accuracy correlates inversely with divergence; therefore, closely-related sequences should be aligned before more distantly related sequences, and radical changes to the joining order will tend to reduce accuracy. In practice, guide trees are often highly imbalanced such that many guide tree nodes join daughter subtrees where one is much larger. The smaller subtree is often a single sequence; Fig. S2 shows an extreme (but not unusual) example where all nodes are $1 : n$, i.e. join a single sequence to all others aligned so far. In such a tree, none of the pair-wise alignments joins two large groups. The construction described here guarantees that large groups are joined close to the root even when the tree is highly unbalanced. When the tree is more balanced, more or all of the joining order is preserved close to the leaves, as illustrated in Fig. S3. When multiple guide tree permutations are applied to the same HMM parameters, e.g. in a stratified ensemble, this design achieves a useful speed optimisation because the alignments of a, b and c can be computed once, leaving only three pair-wise alignments per permutation to complete the MSA. If parameters are perturbed, the guide tree is always recomputed before applying a permutation to account for changes to the EE distance matrix.

Progressive alignment variants compared to previous methods

Neighbour-Joining [3] (N-J) is sometimes used to construct guide trees, e.g. by `ClustalW` [4] and `T-Coffee` [5]. To the extent that more closely related sequences can be aligned more accurately, a UPGMA guide tree tends to give a more accurate progressive alignment because nodes in an N-J tree are predicted evolutionary neighbours, which are not necessarily closest neighbours. I have previously shown that UPGMA guide trees give higher average alignment accuracy than N-J in `Muscle v3` [6].

The heads or tails (`HoT`) method [7] generates variant progressive alignments from a single guide tree by aligning reversed sequences ("tails") at some nodes and unreversed sequences ("heads") at others. It is not obvious that this method will generate any variation, because parameters and the guide tree are held fixed. The pair-wise alignment at a node is usually constructed by dynamic programming which maximises the alignment score (sum of substitution scores and gap penalties), and this score does not change if the alignment is reversed. If an alignment of reversed sequences has the highest score, the equivalent alignment of unreversed sequences will also have the highest score. However, reversed sequences may nevertheless give different alignments due to issues such as floating-point rounding and tie-breaking in cases where more than one alignment has the highest possible score. Any method for generating variants potentially has merit and is worth considering in a larger framework, but it is self-evident that the variations produced by `HoT` can explore only a tiny fraction of the space of equally plausible alignments compared to `Muscle5`.

`Unistrap` [8] induces variant guide trees by changing the order of input sequences to a chosen aligner, e.g. `MAFFT` or `Clustal-Omega`. As with `HoT`, it is not immediately obvious that changes will be induced because the guide tree is calculated from a matrix of all pair-wise distances, and pair-wise distances are the same regardless of input ordering. However, computational artefacts such as tie-breaking may nevertheless cause the guide tree to change. This method has similar limitations to `HoT`: variation in the guide tree may be absent or marginal, and most or all of the ensemble may then reflect consistent bias towards identical or similar guide trees.

`GUIDANCE2` generates variant guide trees starting from an initial MSA generated with a default

guide tree. Re-sampled MSAs are generated by sampling columns with replacement, and a N-J tree is generated from each re-sampled MSA. `HoT` variant alignments are generated from each guide tree. Compared to the `Muscle5` procedure, this has a number of disadvantages. As explained above, there is compelling theoretical and empirical evidence that N-J guide trees are generally inferior to UPGMA trees. There is no lower bound on the variation in guide trees. Systematic bias due to the guide tree in the initial MSA may be reflected in the re-sampled N-J trees and hence propagate to the final ensemble.

`Muscle5` improves on previous methods by using UPGMA, applying minimal modifications consistent with guaranteeing substantial variation in progressive joining order, especially close to the root where progressive bias is most likely to manifest and most likely to degrade phylogenetic tree inference.

Substitution matrix variation compared to previous methods

To the best of my knowledge, all previous methods for generating variant alignments have used a fixed substitution matrix, except for a 1995 study of arthropod phylogeny [9] which assessed the effects of varying the transition-transversion ratio. Here, `Muscle5` introduces an important innovation by varying each substitution score in the matrix independently of the others. This can be motivated and interpreted as follows. Choosing a substitution matrix is equivalent to choosing parameters for an evolutionary model; e.g., a PAM matrix is equivalent to parameters for the JTT model [10]. If the substitution matrix is held fixed, this is equivalent to assuming that all sites evolve according to the same model with the same parameters. Of course, of of these assumptions are highly unrealistic; in fact, constraints vary greatly between sites, and tractable models are drastic simplifications of selective constraints *in vivo*. Even if a supposedly ideal algorithm could optimise per-site parameters simultaneously with likelihoods of the estimated alignment and tree, the model would remain a drastic simplification and the predicted alignment and tree could still be badly wrong. Therefore, rather than striving towards an unattainable "optimal" solution which may be biologically incorrect, it is better to assess the consequences of using the best tractable simplified model. `Muscle5` implements this approach by sampling from a large space of equally good, and hence equally bad, alternative parameters for an unrealistic model. Variations in downstream inferences are necessarily due to errors, and per the central

theme of this work this observation leads immediately to quantitative assessments of uncertainty in those inferences.

Gap penalty variation compared to previous methods

To the best of my knowledge, all previous methods for generating variant alignments use conventional affine gap penalties with one open penalty and one extend penalty. Gap penalties are held fixed in many of these methods, including `WpsBOOT` [11], `HoT` and `Unistrap`. In the *Apicomplexa* study [12], gap open and extension penalties of `ClustalW` were explored over large ranges of values. In `GUIDANCE2`, the gap open penalty is varied, but not the gap extension penalty.

`Muscle5` improves on previous gap penalty variation methods in several respects. The HMM implements double-affine gaps with two open and two extend penalties, giving four adjustable parameters. Double-affine gaps can achieve higher alignment accuracy than single-affine gaps, and explore a larger space of alignments when parameters are varied. A single constant (α) determines the amplitude of variations in all gap and substitution parameters, with a value determined by biologically-motivated constraints. Crucially, `Muscle5` replicates have approximately equal average accuracy superior to state-of-the-art conventional MSA methods on structural benchmarks, while replicates from other methods have degraded average accuracy.

Super5 algorithm

The `Super5` algorithm was designed to scale `PPP` by introducing divide-and-conquer heuristics. A sketch of the algorithm workflow is given in Fig. S4.

Redundancy reduction

The first step of `Super5` aims to identify clusters of two or more highly similar sequences in the input data. For each cluster, a representative sequence is identified. Representatives are propagated for subsequent processing while the remaining sequences are set aside and added back into the representative alignment in the final step. This strategy is effective in reducing computational cost if the number of representatives is substantially smaller than the number of

input sequences, which is often the case in practice. Clusters are constructed using a greedy list removal strategy similar to the `UCLUST` algorithm [13]. Input sequences are sorted by decreasing length, with the goal of ensuring that shorter fragments do not become representatives. A k-mer index on the representatives is used to prioritise sequence comparisons by U-sorting [13] with a maximum of 16 rejections. If an input sequence matches a representative with expected error rate $EE < 0.01$, it is assigned to the corresponding cluster, otherwise it becomes a new representative. This strategy reduces the $O(N^2)$ cost of all-vs-all comparison of N input sequences to an effective cost of $O(NR)$, where R is the number of representatives. This clustering method is called `UCLUST-EE`.

Coarse clustering

The next step divides representatives into clusters small enough to be tractable for `PPP`, i.e. a few hundred sequences. A first-draft set of clusters is obtained by `UCLUST-EE` with $EE < 0.3$. Clusters which are bigger than the maximum size (default 500) are sub-divided by `UCLUST-EE` with $EE < 0.1$. Any remaining clusters which are still too large are sub-divided at random.

Intra-cluster alignment and consensus

Each coarse cluster is aligned by `PPP`, and the consensus sequence for each MSA is calculated by taking the highest-frequency symbol from each column, deleting any positions where this symbol is a gap.

Guide tree construction

An all-vs-all EE distance matrix is calculated from the consensus sequences, and a biased UPGMA tree [6] constructed from the distance matrix.

Representative MSA

MSAs for coarse clusters are combined by progressive alignment following the guide tree. Profile alignment is performed by `PPP-pa` up to a size threshold (2,000 pairs by default), otherwise by `PPP-ps` where the threshold number of sequence pairs is selected at random. This yields an MSA of all representative sequences.

Final MSA

The final MSA is constructed by re-introducing the non-representative sequences set aside in the first step, using the previously constructed pair-wise alignments to their corresponding representatives. These pair-wise alignments imply transitive alignments of non-representative sequences to the representative MSA, which are used to construct the final MSA.

Assessing uncertainty in predictions from simplified evolutionary models

`Muscle5` introduces perturbations into its HMM using a simple, ad hoc procedure which may appear unjustified or unnatural to readers versed in the application of mathematical models to evolution. Here, I offer some comments on conventional approaches and my thinking behind the design of `Muscle5`.

Probabilistic models of evolution are used to predict alignments and/or phylogenetic trees by attempting to maximise likelihood (in a frequentist framework) or posteriors (Bayesian). Conventional wisdom dictates that an ideal algorithm would simultaneously optimise model parameters together with the alignment and tree. However, evolutionary models are not realistic – on the contrary, they are drastic simplifications compared to the complex reality of biological sequence evolution. Even if simultaneous optimisation were tractable, the predicted alignments and trees would surely be prone to errors due to simplifications in the model and the occurrence of low-probability events, and we therefore should not expect that ideally optimised predictions would always or usually be correct. In fact, we should not necessarily even expect that the correct alignment is in the set of possible optimal alignments that are reachable by a given model. This can be illustrated by a toy example. Consider two species X and Y with most recent ancestral sequence `MQTF`. X and Y have independent insertions (`I` and `L` respectively) following the `T`. The correct alignment by residue homology is then (`MQTI-F`, `MQT-LF`) or equivalently (`MQT-IF`, `MQTL-F`), because `I` and `L` are not descended from the same ancestral letter. However, standard pair-wise alignment algorithms will never predict gaps in opposite sequences in adjacent columns because a single substitution has higher probability than two insertions. The predicted alignment will be (`MQTIF`, `MQTLF`), which is not correct. If this alignment is given to a

maximum likelihood tree algorithm, it will incorrectly infer that the (`I`, `L`) column contains a substitution and therefore calculate an incorrect likelihood by the standards of its own model. Finally, I am sceptical that there is enough information in a typical set of input sequences to effectively optimise a model with many parameters, especially if parameters are allowed to vary by site, as should clearly be the case with substitution rates.

I suspect that the best algorithms we have today achieve (in)accuracy comparable to that which would be achieved by a hypothetical ideal algorithm, in which case a point of diminishing returns has been reached from a biological perspective (as opposed to a computer science perspective where the optimisation problem is interesting on its own terms). In the design of `Muscle5`, I therefore explicitly rejected the goal of improved optimisation and instead sought to determine the sensitivity of predicted alignments to perturbations in model parameters as a proxy for assessing uncertainty arising from the use of simplified models and the likely presence of unlikely events [*sic*] in more challenging cases.

Balifam benchmark

I implemented a new structure-based benchmark, `Balifam`, to evaluate accuracy on large datasets of up to 10,000 sequences. `Balifam` was constructed from reference alignments in `Balibase v3` [14] by adding homologs identified by `PFAM` [15] (Fig. S5). Each reference sequence in `Balibase` was aligned to `PFAM`. For each `PFAM` domain identified in a given `Balibase` reference alignment, the subset of columns in the reference which aligned to that domain were extracted, giving a core alignment. For each domain, the core alignment with largest number of sequences was chosen. (This step was necessary because `Balibase` is highly redundant, often re-using the same sequences in different sets). To each core alignment, I added homologs from the corresponding `PFAM` "full" alignment. The `PFAM` alignments *per se* were not used; this procedure served only to increase the size of the datasets. Random subsets of size 100, 1,000 and 10,000 respectively were selected and added to the core alignments, yielding 59 sets in `Balifam-100`, 56 sets in `Balifam-1000` and 36 sets in `Balifam-10000`. Alignment accuracy is assessed on the subset of sequences in the `Balibase` alignment. Benchmark data is available at `https://github.com/rcedgar/balifam`. `ProbCons` 1.0 and `MUSCLE` v3 failed on Balifam as they

do not scale to large datasets.

Accuracy results on Balifam

Tests were run on `c5a.4xlarge` instances. On `Balifam-10000`, `Muscle5` aligns 59% of columns correctly, which is a 13% improvement over `Clustal-omega` (52% columns correct) and a 26% improvement over `MAFFT` (47% columns correct). This difference is significant by the Wilcoxon test: `Super5` > `Clustal-omega` with $p = 1.2 \times 10^{-4}$ and `Super5` > `MAFFT` with $p = 1.4 \times 10^{-7}$. On Balifam, ensembles generated by `Muscle5` aligned an average of 59% of columns correctly, 13% better than `Clustal-omega` (52% correct) and 26% better than `MAFFT` (47% correct).

RdRp alignment and tree

I re-aligned the 4,617 RNA-dependent RNA polymerase (RdRp) sequences from Wolf2018 using the Super5 algorithm, generating a stratified ensemble. I reduced replicate MSAs to a tractable size ($n = 250$ sequences) for maximum-likelihood tree estimation by constructing the $none.0$ alignment of all sequences, estimating an approximate tree T_{FT} by `FastTree` [16], identifying a set of edges which divide T_{FT} into n subtrees of approximately size, and selecting one representative sequence at random from each subtree; a similar protocol was used by Wolf2018. Representative sequences were extracted from the stratified ensemble and corresponding maximum-likelihood trees were estimated by RaxML. Varying the random number seed d for representative sequence selection provides another mechanism for generating replicate MSAs and hence replicate trees; these are denoted $d.perm.s$. Phylum branching order was inferred by identifying the best-fit subtree for each phylum (Methods); bootstrap confidences were assigned from the highest-confidence edges linking phylum subtrees. Following Wolf2018, trees were estimated using `RAxML` [17] and confidence was measured by transfer expectation.

References

1. Holmes, I. & Durbin, R. Dynamic programming alignment accuracy. *Journal of computational biology* **5,** 493–504 (1998).
2. Do, C. B., Mahabhashyam, M. S., Brudno, M. & Batzoglou, S. ProbCons: Probabilistic consistency-based multiple sequence alignment. *Genome research* **15,** 330–340 (2005).
3. Saitou, N. & Nei, M. The neighbor-joining method: a new method for reconstructing phylogenetic trees. *Molecular biology and evolution* **4,** 406–425 (1987).
4. Thompson, J. D., Higgins, D. G. & Gibson, T. J. CLUSTAL W: improving the sensitivity of progressive multiple sequence alignment through sequence weighting, position-specific gap penalties and weight matrix choice. *Nucleic acids research* **22,** 4673–4680 (1994).
5. Notredame, C., Higgins, D. G. & Heringa, J. T-Coffee: A novel method for fast and accurate multiple sequence alignment. *Journal of molecular biology* **302,** 205–217 (2000).
6. Edgar, R. C. MUSCLE: multiple sequence alignment with high accuracy and high throughput. *Nucleic acids research* **32,** 1792–1797 (2004).
7. Landan, G. & Graur, D. Heads or tails: a simple reliability check for multiple sequence alignments. *Molecular biology and evolution* **24,** 1380–1383 (2007).
8. Chatzou, M., Floden, E. W., Di Tommaso, P., Gascuel, O. & Notredame, C. Generalized bootstrap supports for phylogenetic analyses of protein sequences incorporating alignment uncertainty. *Systematic Biology* **67,** 997–1009 (2018).
9. Wheeler, W. C. Sequence alignment, parameter sensitivity, and the phylogenetic analysis of molecular data. *Systematic Biology* **44,** 321–331 (1995).
10. Jones, D. T., Taylor, W. R. & Thornton, J. M. The rapid generation of mutation data matrices from protein sequences. *Bioinformatics* **8,** 275–282 (1992).
11. Chang, J.-M. *et al.* Incorporating alignment uncertainty into Felsenstein's phylogenetic bootstrap to improve its reliability. *Bioinformatics* **37,** 1506–1514 (2021).
12. Morrison, D. A. & Ellis, J. T. Effects of nucleotide sequence alignment on phylogeny estimation: a case study of 18S rDNAs of Apicomplexa. *Molecular biology and evolution* **14,** 428–441 (1997).

13. Edgar, R. C. Search and clustering orders of magnitude faster than BLAST. *Bioinformatics* **26,** 2460–2461 (2010).

14. Thompson, J. D., Plewniak, F. & Poch, O. BAliBASE: a benchmark alignment database for the evaluation of multiple alignment programs. *Bioinformatics (Oxford, England)* **15,** 87–88 (1999).

15. Bateman, A. *et al.* The Pfam protein families database. *Nucleic acids research* **32,** D138–D141 (2004).

16. Price, M. N., Dehal, P. S. & Arkin, A. P. FastTree 2–approximately maximum-likelihood trees for large alignments. *PloS one* **5,** e9490 (2010).

17. Stamatakis, A. RAxML version 8: a tool for phylogenetic analysis and post-analysis of large phylogenies. *Bioinformatics* **30,** 1312–1313 (2014).

Supplementary Tables and Figures

Term	Description
Ensemble	Collection of alternative MSAs of the same sequences.
H-ensemble	Ensemble of high-accuracy MSAs such that no particular MSA from this ensemble (or by any other method) is preferred *a priori*.
Stratified ensemble	H-ensemble constructed to have subsets (strata) where a potential source of systematic errors is held fixed, e.g. guide tree topology or gap penalty values. Enables detection of particular types of bias / systematic error in an inference made from an MSA.
Diversified ensemble	H-ensemble constructed to maximize diversity in biases of the alternative alignments, especially systematic errors.
H-ensemble confidence	(HEC) Confidence estimate for an inference derived from an MSA. Range 0...1. Calculated as the fraction of MSAs in a diversified H-ensemble which support the inference. Measures robustness of the inference against errors / ambiguities / uncertainties in the MSA.
Column confidence	(CC) HEC of an alignment column. Provides an unsupervised estimate of the probability that column is correct. If CC is close to 1, the column is reproduced in most MSAs in the ensemble and the column is likely to be correct.
Alignment confidence	(AC) Mean CC for an MSA. Correlates with the number of errors in the MSA. If AC is close to 1, this indicates high accuracy.
Mean alignment confidence	(MAC) Mean AC for MSAs in an ensemble. If MAC is close to 1, the alignments are robust and the error rate is probably low.
Topology confidence	(TC) HEC of a tree topology.
Edge confidence.	(EC) HEC of a tree edge.
Monophyly	(m) Measure of monophylicity on one tree in range 0...1. Calculated as m = TP/(TP + FP + FN) for the best-fit subtree of a group. If m=1, the group is perfectly monophyletic, if m<1 it is polyphyletic.
Ensemble Monophyly	(EM) Mean monophyly of a group over an ensemble. If EM is close to 1, this indicates high confidence in the monophylicity of the group.

Table S1. Terminology.

Summary of technical terms.

	Balibase (protein)			Bralibase (RNA)		
Method	Time (mins.)	Max mem (Mb)	TC	Time (mins.)	Max mem (Mb)	TC
Clustal-Omega	5	0.98	0.56	5	0.5	0.73
MAFFT	35	0.5	0.61	7	0.4	0.73
MUSCLE v5	27	2.1	0.63	6	0.8	0.78
MUSCLE v3	8	0.08	0.53	2	0.2	0.75
PROBCONS	182	0.54	0.62	n/a	n/a	n/a

Table S2. Balibase and Bralibase results for Clustal-Omega, MUSCLE, MAFFT and ProbCons.

Comparison of MUSCLE versions 3 and 5 against Clustal-Omega v1.2.4, MAFFT v7.5 and ProbCons v1.0. Time (mins) is total elapsed time, maximum memory is given in Mb. Accuracy is measured by fraction of predicted columns that are correct (TC).

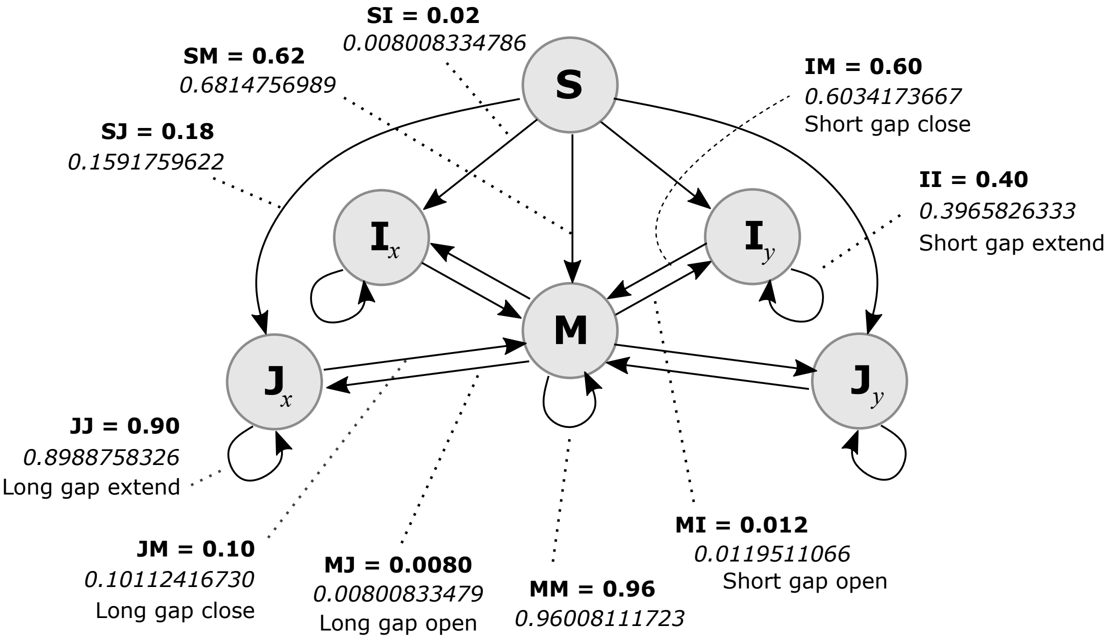

Figure S1. Hidden Markov model.

HMM used by the `Muscle5` algorithm, showing transition probabilities, which are equivalent to gap penalties. There are two insert states (I and J) for each sequence . Default probabilities for `Muscle5` are shown in bold; defaults for `ProbCons` are italic. `ProbCons` values are specified to 10 significant figures per its source code, while `Muscle5` values are rounded to two significant figures on the premise that small differences should be immaterial to alignment quality (which is validated *post-hoc* by benchmark results). Emission probabilities are taken from the joint-probability form of the BLOSUM62 matrix (not shown).

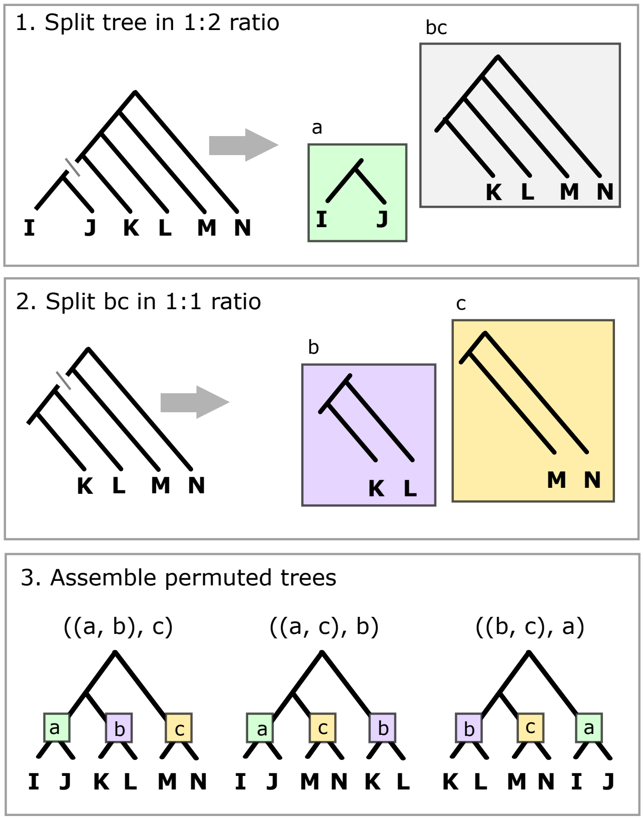

Figure S2. Guide tree permutations.

1. The guide tree is split by identifying an edge which divides the tree into subtrees *a* and *bc* with approximately one third and two thirds of the leaves, respectively. 2. Subtree *bc* is split into *b* and *c* with approximately equal sizes. 3. Permuted trees are assembled. In this example, the tree is maximally unbalanced

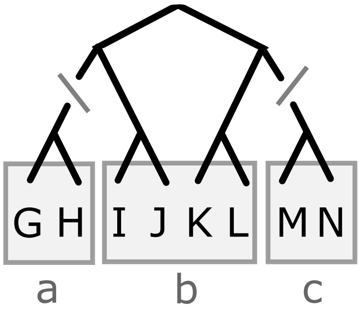

Figure S3. Dividing a balanced tree.

Example of splitting a balanced tree into three approximately equal-sized subtrees. Note that all pair-wise leaf alignments are preserved in all permutations, in contrast to the example in Fig. S2 where $I + J$ is preserved but the order is changed for all other leaves, showing that a maximally unbalanced tree is a worst-case scenario for this approach.

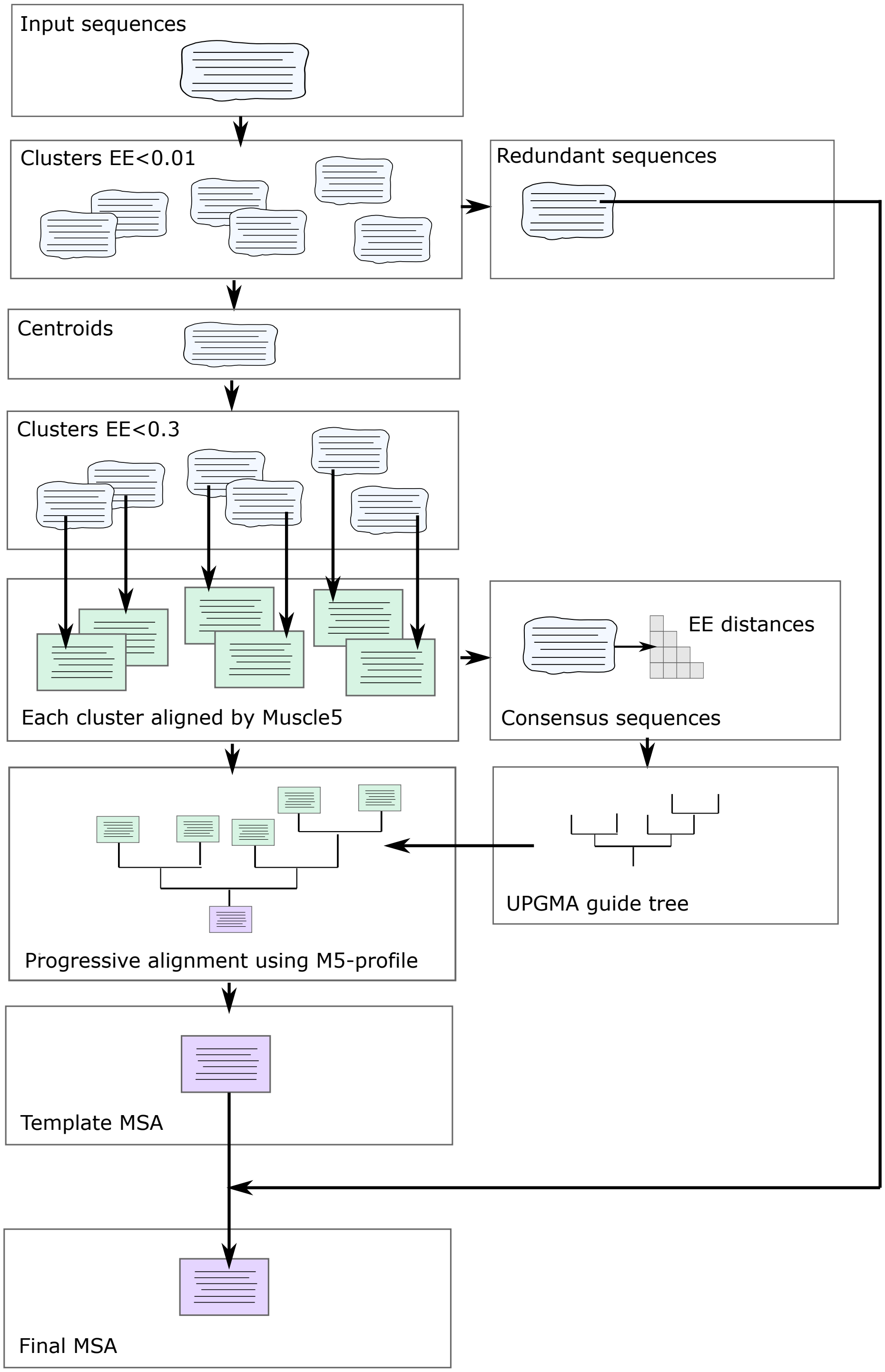

Figure S4. Workflow of the `Super5` algorithm.

`Super5` applies a divide-and-conquer strategy to `PPP`, enabling scaling to larger datasets.

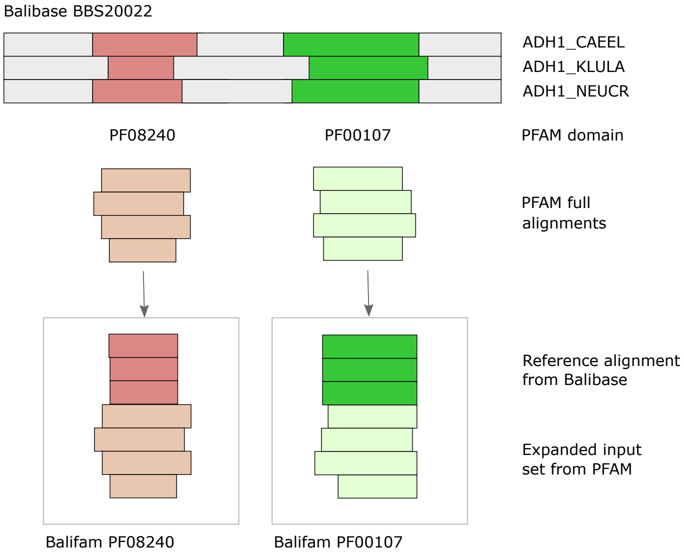

Figure S5. Construction of `Balifam`.

`Balifam` is constructed by using `PFAM` to identify domains in `Balibase` reference alignments. Here, three of the 58 sequences in reference set `BBS20022` are shown as examples. These sequences contain two domains, `PF08240` Alcohol dehydrogenase GroES-like domain and `PF00107` Zinc-binding dehydrogenase. Columns from the reference alignment matching each domain are extracted and combined with unaligned sequences from the corresponding `PFAM` full alignments.

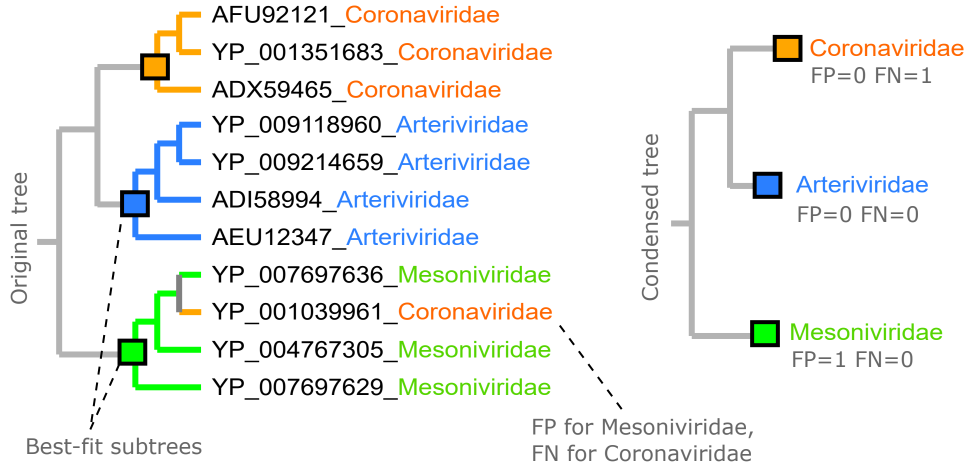

Figure S6. Condensing a tree and calculating monophyly.

This hypothetical example shows how a tree is condensed from species to family. Leaves are species, belonging to three families: *Coronaviridae*, *Arteriviridae* and *Mesoniviridae*. For each family, the best fit subtree is identified as the node which gives fewest errors (FP+FN). *Arteriviridae* is monophyletic (no errors), but one coronavirus species is misplaced inducing a FP for *Mesoniviridae* and a FN for *Coronaviridae*. The tree is condensed by deleting all edges under the best-fit subtree nodes and labeling these nodes, which are now leaves, with the family names. This simplifies the tree and eliminates the error. Monophyly is defined as $m = TP/(TP + FP + FN)$. In this example, *Arteriviridae* has $TP = 4, FP = 0, FN = 0$ hence $m = 0$, *Coronaviridae* has $TP = 3$, $FP = 0$, $FN = 1$, $m = 3/4 = 0.75$ and *Mesoniviridae* has $TP = 3$, $FP = 1$, $FN = 0$, $m = 3/4 = 0.75$. For calculating monophyly of a higher rank, e.g. phylum, errors at the lower rank used for assessment (e.g. class) are not counted; rather, the tree is first condensed to class, then phylum monophyly is assessed using the best-fit nodes for class.
